# Supplementary figures and images for: Basal sauropodomorph locomotion: ichnological lessons from the Late Triassic trackways of bipeds and quadrupeds (Elliot Formation, main Karoo Basin)
Source: PeerJ. 2023 Sep 28;11:e15970. doi: 10.7717/peerj.15970 (PMC10542822; doi:10.7717/peerj.15970)

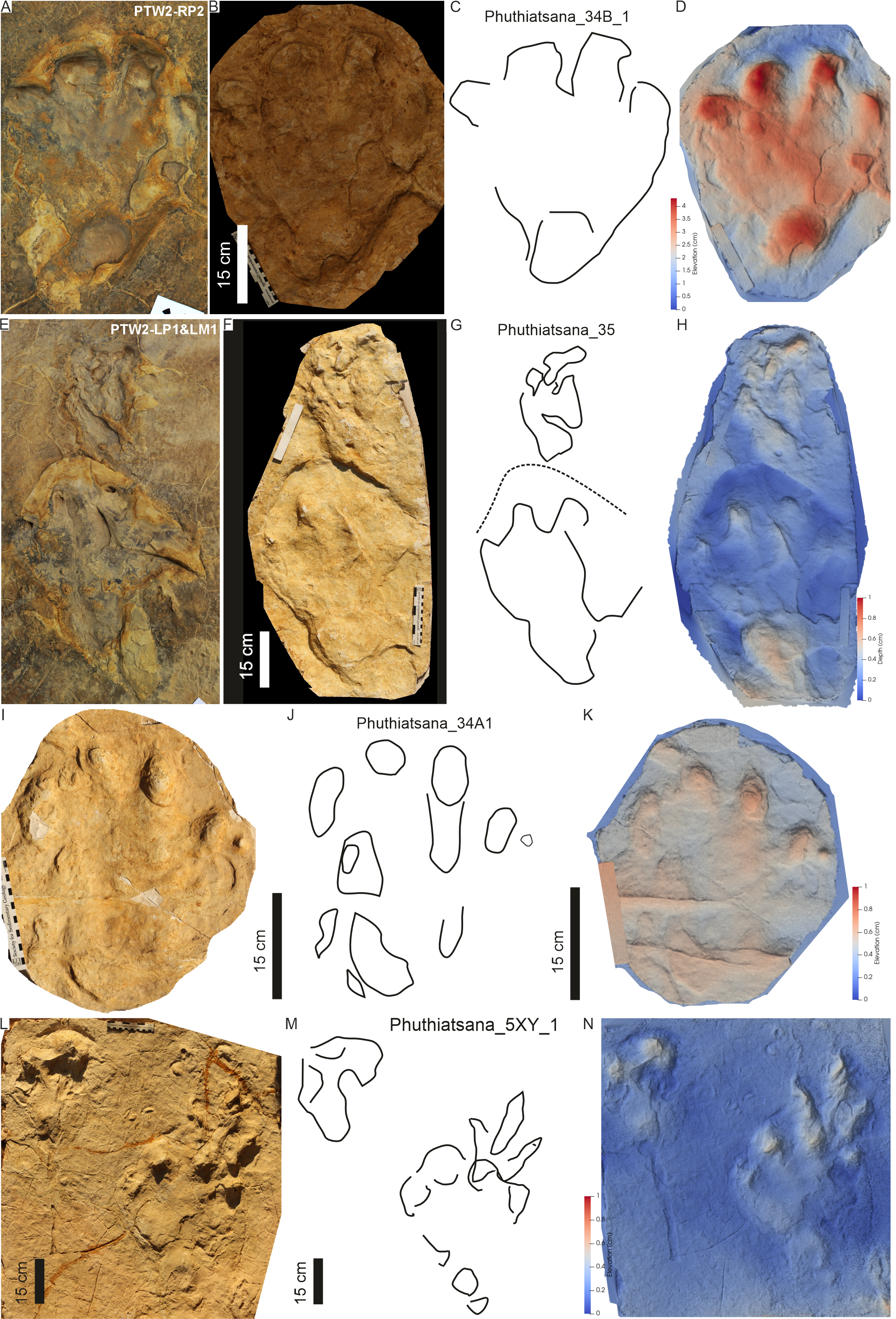

Supplement: Supplemental Information 1 — (a) Trackway ‘PT2’ right pes (RP2) as photographed in the field. (b) Ellenberger cast of right pes from (a) labelled “Phuthiatsana_34B_1" in collections (P. jaquesi). (c) Interpretative outline drawing from the cast in (b). (d) False colour depth map of cast. (e) Trackway ‘PT2’ left pes-manus pair (LP1, LM1) as photographed in the field. (f) Ellenberger cast left pes-manus pair in (e) labelled “Phuthiatsana_35" in collections (P. jaquesi). (g) Interpretative outline drawing from the cast in (f). (h) False colour depth map of cast. (i) Ellenberger cast “Phuthiatsana_34A1" from collections but unknown from the Phutiastana site. (j) interpretative outline drawing. (k) False colour depth map of cast. (l) Ellenberger cast “Phuthiatsana_5XY_1" of pes-manus pair and tridactyl track from unknown trackway at Phuthiatsana, (m) Interpretative drawing. (n) False colour depth map of cast. [file peerj-11-15970-s001.png]

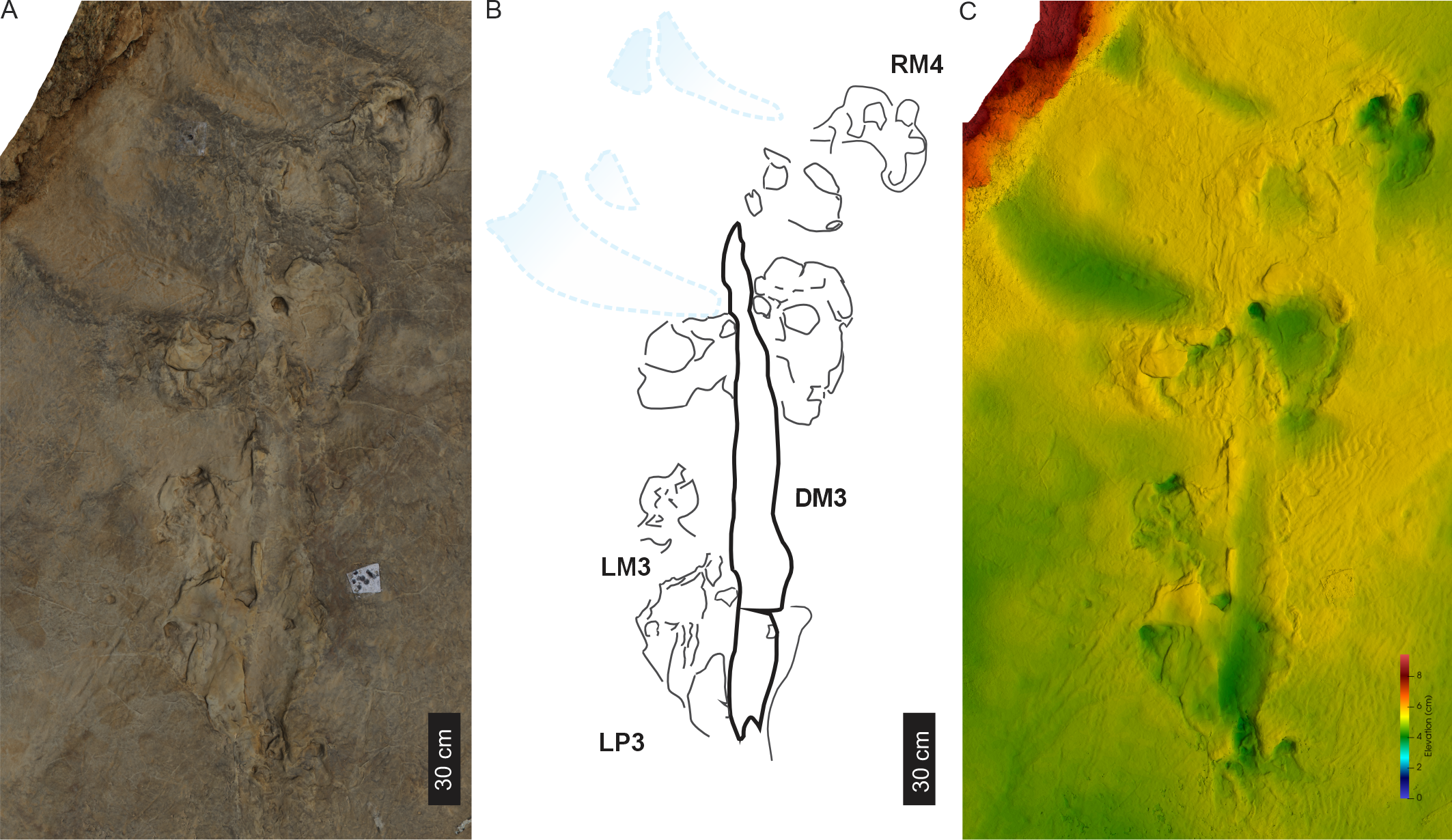

Supplement: Supplemental Information 2 — (a) Orthophotograph, (b) line-drawing overlay, and (c) False-colour depth map of drag trace (DM3) between the penultimate manus track (RM4) and the PT2-LP3 pes-manus pair. Note small, grooved channels (infilled in pale grey) that run across PT2-RP6 footprint. [file peerj-11-15970-s002.png]
